# Case of recurrence of spiradenoma in palpebral conjunctiva

**DOI:** 10.1186/1471-2415-14-85

**Published:** 2014-06-27

**Authors:** Shinya Oie, Akira Sawada, Kiyofumi Mochizuki, Kozue Tsuji, Yoshinobu Hirose, Chiemi Saigo, Hiroshi Yoshikawa

**Affiliations:** 1 Department of Ophthalmology, Gifu University Graduate School of Medicine, 1-1 Yanagido, Gifu-shi 501-1194, Japan; 2 Department of Pathology, Gifu University School of Medicine, 1-1 Yanagido, Gifu-shi 501-1194, Japan; 3 Department of Ophthalmology, Graduate School of Medical Science, Kyushu University, Fukuoka 812-8582, Japan

**Keywords:** Spiradenoma, Sweat gland, Eyelids, Repeated recurrences, Immunohistochemistry

## Abstract

**Background:**

To report a rare case of a recurrence of spiradenoma that developed in the upper eyelid.

**Case presentation:**

A 49-year-old woman who had a second recurrence of a tumor in the right palpebral conjunctiva underwent local resection of the lesion with adjunctive cryotherapy to the surgical site. The tumor consisted of smooth, round to oval nodular lesions approximately 1–3 mm in size with enlarged blood vessels. Histopathologically, the solid and well-circumscribed nodule was located beneath the conjunctival epithelium. It was composed of cells with slightly basophilic-to-clear cytoplasm and round-to-oval nuclei arranged in a trabecular pattern. Periodic acid-Schiff stain was positive in the cytoplasm, and the staining disappeared after digesting by diastase. Many cells in mitosis were observed throughout the tumor but no necrotic cells. Immunohistochemistry showed that the Ki-67 labeling index was 12%. From these findings, we diagnosed this tumor as a recurrence of the spiradenoma. There has been no recurrence and no signs of malignancy in the 6 months after the surgical excision.

**Conclusion:**

Our findings indicate that a spiradenoma should be completely excised surgically because of malignant transformation after repeated recurrences.

## Background

Tumors arising from the sweat glands of the eyelids are uncommon, and the differences in their structure have given rise to much confusion [1-4]. A spiradenoma is a type of sweat gland tumor which has a comparatively good clinically course although it appears to be malignant [1,5]. A spiradenoma involving the eye or eyelid is rare [2,3]. We describe our findings in a patient with a recurrence of spiradenoma that developed in the upper eyelid.

## Case presentation

**Figure 1:**
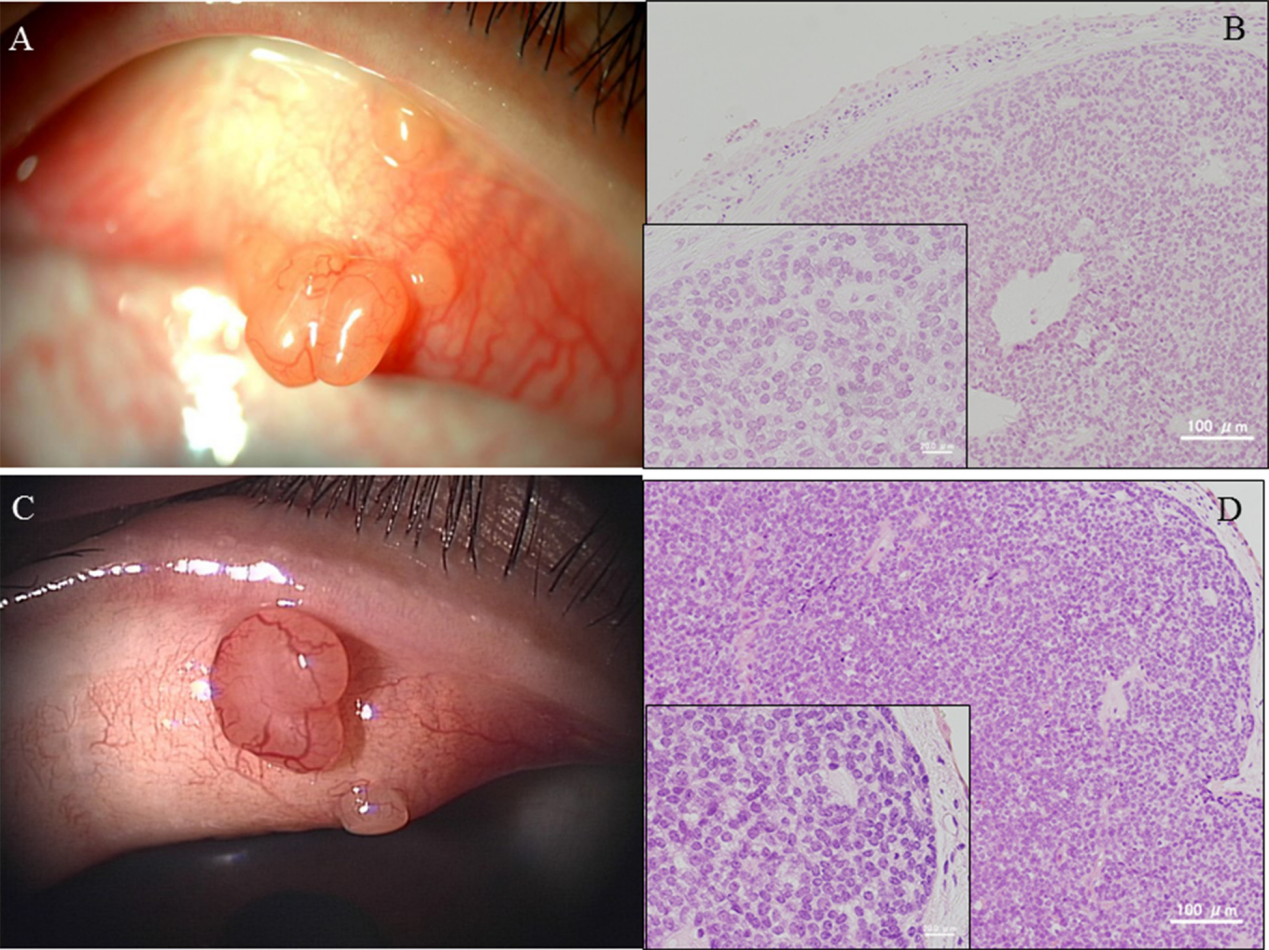
**Clinical appearance of a spiradenoma in primary (A and B) and secondary tumors (C and D). A**. A round to oval-shaped nodule with smooth borders was present in the right upper palpebral conjunctiva. **B**. The tumor cells were arranged in intertwining bands just beneath the conjunctiva. Two types of neoplastic cells were recognized; one had a small dark nucleus representing an undifferentiated cell, and the other was located at the center of the bands with large pale nucleus. Levels of mitotic counts were low (0.3/10 HPF). Hematoxylin-eosin staining. **C**. The tumors can be seen on the right upper palpebral conjunctiva, which was the slightly upper location of the previous tumor. Its surface is smooth. **D**. A solid and well-circumscribed nodule can be seen beneath the conjunctival epithelium. It is composed of cells with slightly basophilic-to-clear cytoplasms and round-to-oval nuclei and arranged in a trabecular pattern. Mitotic activity was increased (5.5/10 HPF) throughout the tumor with no necrosis. Hematoxylin-eosin staining.

**Figure 2:**
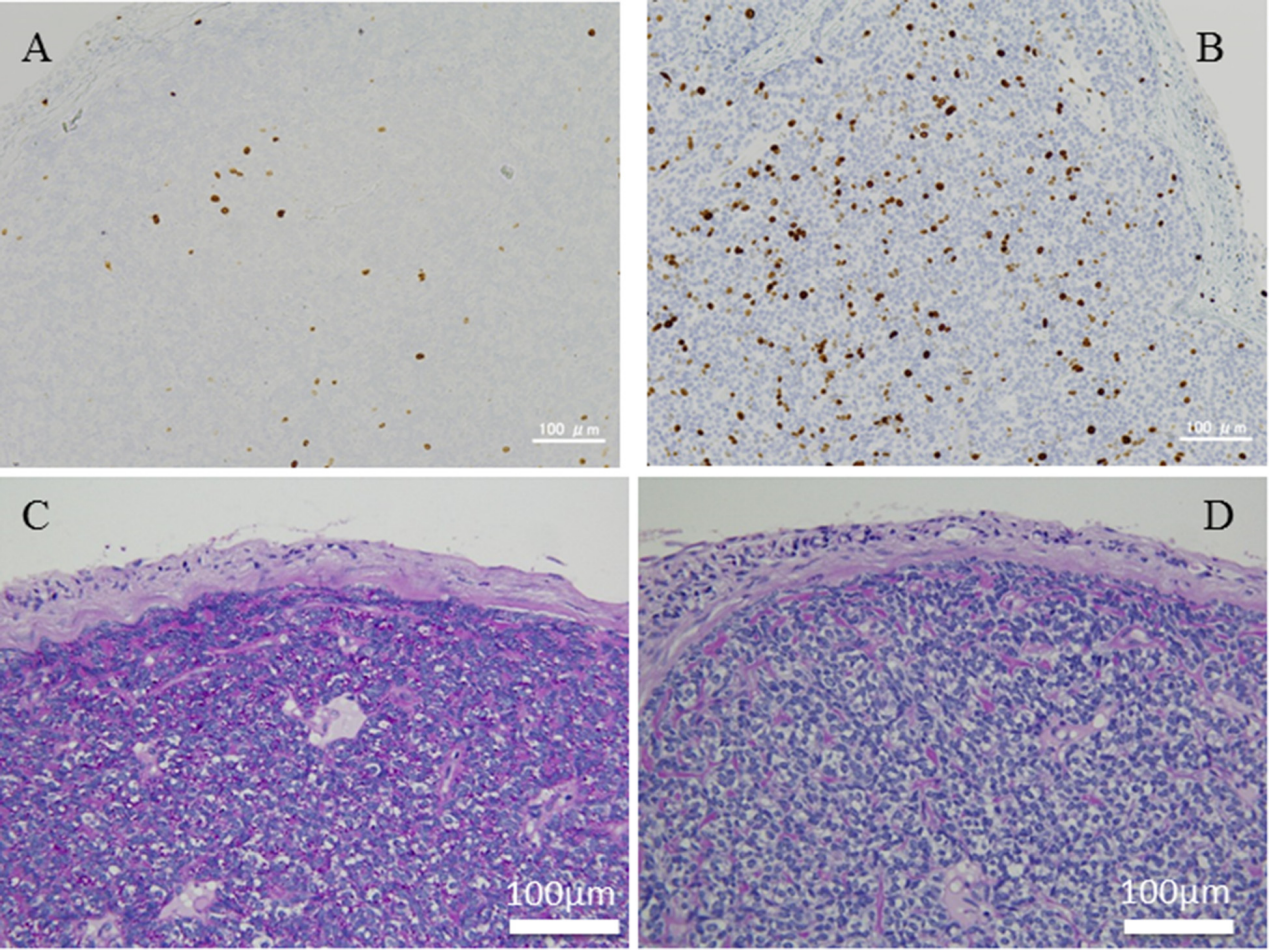
**Histopathology and immunohistochemical staining of a spiradenoma in primary (A) secondary tumors (B, C, and D). A**. The Ki-67 labeling index was 2.8%. **B**. The Ki-67 labeling index was 12%. **C** and **D**. Periodic acid-Schiff staining is positive in the cytoplasm **(B)**, which was digested by diastase **(C)**.

A 49-year-old woman visited a neighborhood eye clinic complaining of a foreign body sensation and pain in her right upper eyelid of several days duration. She was referred to our hospital for further evaluation and treatment on the next day. Several years earlier, she had a tumor excised from her right eyelid, and the tumor was diagnosed as a spiradenoma. Our examination showed that her best-corrected visual acuity (BCVA) was 20/30 OD and 20/20 OS. A round to oval-shaped nodule with smooth borders was present in the right upper palpebral conjunctiva (Figure 1A), which was thought to be the same location of the previous tumor. The tumor was excised and evaluated histopathologically. The tumor cells were arranged in intertwining bands just beneath the conjunctiva. Two types of neoplastic cells were recognized; one had a small dark nucleus representing an undifferentiated cell, and the other was located at the center of the bands with large pale nucleus (Figure 1B). Levels of mitotic counts were low (0.3/10 HPF) and immunohistochemical analysis showed that the Ki-67 labeling index was 2.8% (Figure 2A). It was unclear if there were the tumor cells still in the incisional margin of the slices.One year later, she visited our hospital again complaining of pain in the right eye. A nodule of approximately 1–3 mm in size accompanying by enlarged blood vessels was observed at the right upper palpebral conjunctiva (Figure 1C). This nodule was regarded as a recurrence. Preauricular and cervical lymphadenopathy was not present. Laboratory data including blood chemistry, and serum level of tumor markers were within the normal range. No abnormal findings were seen in the chest X-rays. The tumor was excised with adjunctive cryotherapy and examined histopathologically. The solid and well-circumscribed nodule was located beneath the conjunctival epithelium. The tumor cells were slightly basophilic with clear cytoplasm and oval nuclei were arranged in a thick trabecular pattern (Figure 1D). The two cell pattern, which was recognized in the previous tumor, was inconspicuous. Mitotic activity was increased (5.5/10 HPF) throughout the tumor with no necrosis (Figure 1D). Immunohistochemistry showed a Ki-67 labeling index of 12.0% (Figure 2B). The tumor stained weak positive for epithelial membrane antigen (EMA), and negative for carcinoembryonic antigen (CEA), low molecular-weight cytokeratin (CAM5.2), synaptophysin, and chromogranin A immunohistochemically. Periodic acid-Schiff (PAS) stain was positive in the cytoplasm (Figure 2C), but the positivity disappeared after digestion by diastase (Figure 2D). The tumor was diagnosed as a recurrence of the spiradenoma. There has been no recurrence in the 6 months after the surgical removal and cryotherapy.

## Discussion

A spiradenoma is a tumor of the eccrine sweat glands that arises as a solitary, intradermal, and painful nodule on the chest or face and most frequently in early adulthood. Occasionally, there may be multiple tumors [1,2,5]. It has been well-characterized in the dermatological literature but not in the ophthalmological literature probably because it is uncommon in the eyelid [2,3]. To the best of our knowledge, there has been no report of a primary spiradenoma developing in the palpebral conjunctiva. In our case, the tumor was considered to be a secondary spread of the original tumor at the primary site in the palpebral conjunctiva of the eyelid. The majority of spiradenoma have a benign course [2], and local recurrences have not been reported. Considering our lesion was a local recurrence because of an inadequate surgical removal, we treated the site of the tumor excision with adjunctive cryotherapy to prevent further recurrences.

Immunohistochemical study demonstrated that cytokeratin expression in eccrine spiradenoma was compared with expression in normal eccrine glands and the tubular structures are CEA positive [6,7]. In the present case, the CAM5.2, and CEA were negative in the tumor cells and the EMA was weakly positive. The immunohistochemical results suggest that the tumor may inadequate differentiate towards both the ductal and secretory segments of the eccrine sweat glands.

The differential diagnosis includes cylindroma, hidradenoma and sebaceoma. Dermal cylindroma and spiradenoma in the same patient or in the same tumor mass are well-represented in the literature [3,8]. Cylindroma differs from spiradenoma by displaying islands of cells rimmed by a basement membrane matrix and the presence of few lymphocytes [8]. Immunohistochemical reactivity for CAM5.2, CEA, EMA, S-100, and vimentin in hidradenoma is characteristic [9]. Because of the PAS-positivity and adipophilin expression, our tumor mimicked a low grade malignant tumor including sebaceous differentiations. Sebaceous differentiations is defined as the presence of multiple cytoplasmic fat vacuoles and the positive staining of tumor cells for EMA and negative staining for CEA [10,11].

Malignant changing of spiradenomas is unusual, and the histology of malignant spiradenomas resembles squamous metaplasia or very poorly differentiated pseudosarcomatous spindle cell elements [5]. In cases of a malignant transformation, the patient might die of systemic metastases several months after the diagnosis [5]. Because our tumor had atypical cells with mitotic activity increasing (from 0.3/10 to 5.5/10 HPF) and had a trabecular or alveolar pattern, it appeared to have a more aggressive pattern. The Ki-67 labeling index that is related to the prognostic features was 12% in our case. The tumor contained PAS-positive material, which was identified as glycogen by its breakdown by diastase. Thus, we diagnosed the tumor as a recurrence spiradenoma from the clinical and pathological features of the tumor and might be progressing to malignant changes.

## Conclusions

We describe a recurrence of spiradenoma due to an inadequate surgical excision at a rare site, the palpebral conjunctiva. Although a spiradenoma is usually benign, it may transform to a malignant spiradenoma, and thus we are following this patient carefully.

## Consent

The study was approved by the Ethics Committees of Gifu University Hospital. Written informed consent was obtained from the patient for publication of this Case report and any accompanying images. A copy of the written consent is available for review by the Series Editor of this journal.

## Competing interests

The authors have no proprietary or financial interest in any products used in this study.

## Authors’ contributions

Conceiving and designing the study (MK); data collection (OS, TK, HY); interpreting the data (SA, MK); writing the manuscript (SA), providing critical revisions (HY, YH) and approving the final version (OS, SA, MK, TK, HY, YH). All authors read and approved the final manuscript.

## Pre-publication history

The pre-publication history for this paper can be accessed here:

http://www.biomedcentral.com/1471-2415/14/85/prepub
